# Comparative Effectiveness of Sodium-Glucose Cotransporter 2 Inhibitors vs Sulfonylureas in Patients With Type 2 Diabetes

**DOI:** 10.1001/jamainternmed.2021.2488

**Published:** 2021-06-28

**Authors:** Yan Xie, Benjamin Bowe, Andrew K. Gibson, Janet B. McGill, Geetha Maddukuri, Ziyad Al-Aly

**Affiliations:** 1 Clinical Epidemiology Center, Research and Development Service, VA St Louis Health Care System, St Louis, Missouri; 2 Department of Epidemiology and Biostatistics, College for Public Health and Social Justice, St Louis University, St Louis, Missouri; 3 Veterans Research and Education Foundation of St Louis, St Louis, Missouri; 4 Department of Medicine, Washington University School of Medicine in St Louis, St Louis, Missouri; 5 Nephrology Section, Medicine Service, VA St Louis Health Care System, St Louis, Missouri; 6 Institute for Public Health, Washington University in St Louis, St Louis, Missouri

## Abstract

**Importance:**

In the treatment of type 2 diabetes, evidence of the comparative effectiveness of sodium-glucose cotransporter 2 (SGLT2) inhibitors vs sulfonylureas—the second most widely used antihyperglycemic class after metformin—is lacking.

**Objective:**

To evaluate the comparative effectiveness of SGLT2 inhibitors and sulfonylureas associated with the risk of all-cause mortality among patients with type 2 diabetes using metformin.

**Design, Setting, and Participants:**

A cohort study used data from the US Department of Veterans Affairs compared the use of SGLT2 inhibitors vs sulfonylureas in individuals receiving metformin for treatment of type 2 diabetes. A total of 23 870 individuals with new use of SGLT2 inhibitors and 104 423 individuals with new use of sulfonylureas were enrolled between October 1, 2016, and February 29, 2020, and followed up until January 31, 2021.

**Exposures:**

New use of SGLT2 inhibitors or sulfonylureas.

**Main Outcomes and Measures:**

This study examined the outcome of all-cause mortality. Predefined variables and covariates identified by a high-dimensional variable selection algorithm were used to build propensity scores. The overlap weighting method based on the propensity scores was used to estimate the intention-to-treat effect sizes of SGLT2 inhibitor compared with sulfonylurea therapy. The inverse probability of the treatment adherence weighting method was used to estimate the per-protocol effect sizes.

**Results:**

Among the 128 293 participants (mean [SD] age, 64.60 [9.84] years; 122 096 [95.17%] men), 23 870 received an SGLT2 inhibitor and 104 423 received a sulfonylurea. Compared with sulfonylureas, SGLT2 inhibitors were associated with reduced risk of all-cause mortality (hazard ratio [HR], 0.81; 95% CI, 0.75-0.87), yielding an event rate difference of −5.15 (95% CI, −7.16 to −3.02) deaths per 1000 person-years. Compared with sulfonylureas, SGLT2 inhibitors were associated with a reduced risk of death, regardless of cardiovascular disease status, in several categories of estimated glomerular filtration rate (including rates from >90 to ≤30 mL/min/1.73 m^2^) and in participants with no albuminuria (albumin to creatinine ratio [ACR] ≤30 mg/g), microalbuminuria (ACR >30 to ≤300 mg/g), and macroalbuminuria (ACR >300 mg/g). In per-protocol analyses, continued use of SGLT2 inhibitors was associated with a reduced risk of death compared with continued use of sulfonylureas (HR, 0.66; 95% CI, 0.60-0.74; event rate difference, −10.10; 95% CI, −12.97 to −7.24 deaths per 1000 person-years). In additional per-protocol analyses, continued use of SGLT2 inhibitors with metformin was associated with a reduced risk of death compared with SGLT2 inhibitor treatment without metformin (HR, 0.70; 95% CI, 0.50-0.97; event rate difference, −7.62; 95% CI, −17.12 to −0.48 deaths per 1000 person-years).

**Conclusions and Relevance:**

In this comparative effectiveness study analyzing data from the US Department of Veterans Affairs, among patients with type 2 diabetes receiving metformin therapy, SGLT2 inhibitor treatment was associated with a reduced risk of all-cause mortality compared with sulfonylureas. The results provide data from a real-world setting that might help guide the choice of antihyperglycemic therapy.

## Key Points

**Question:**

What is the comparative effectiveness of sodium-glucose cotransporter 2 inhibitors vs sulfonylureas associated with the risk of all-cause mortality among individuals using metformin for treatment of type 2 diabetes?

**Findings:**

In this comparative effectiveness study analyzing data from the US Department of Veterans Affairs and including 128 293 individuals with type 2 diabetes receiving metformin, use of sodium-glucose cotransporter 2 inhibitors was associated with reduced risk of all-cause mortality compared with sulfonylureas, regardless of cardiovascular disease status, estimated glomerular filtration rate category, and albuminuria status. Use of sodium-glucose cotransporter 2 inhibitors with metformin therapy was associated with a reduced risk of all-cause mortality compared with sodium-glucose cotransporter 2 inhibitors without metformin therapy.

**Meaning:**

The results of this cohort study provide real-world data on the risk of all-cause mortality associated with sodium-glucose cotransporter 2 inhibitors vs sulfonylureas, which may help guide the choice of antihyperglycemic therapy in people with type 2 diabetes.

## Teaser

This cohort study compares the risk of all-cause mortality in individuals using sodium-glucose cotransporter 2 inhibitors vs sulfonylureas in combination with metformin for treatment of type 2 diabetes.

## Introduction

The introduction of sodium-glucose cotransporter 2 (SGLT2) inhibitors as a new class of antihyperglycemics that reduces the risk of adverse cardiovascular and kidney events has been a welcome addition to the armamentarium of therapeutics in diabetes.^1,2,3,4,5,6,7^ Evidence also suggests that the beneficial effects of SGLT2 inhibitors extend to people without diabetes.^8,9,10^ However, randomized clinical trials of SGLT2 inhibitors examined the effect of SGLT2 inhibitors vs placebo; the trials did not provide head-to-head comparison with other second-line antihyperglycemic agents.^1,2,3^ Several large, real-world studies provided evidence on the use of SGLT2 inhibitors vs dipeptidyl peptidase-4 inhibitors and SGLT2 inhibitors vs other antihyperglycemics on cardiovascular and kidney outcomes.^11,12,13,14,15,16,17,18,19,20,21,22^ However, comparative data from real-world settings on SGLT2 inhibitors vs sulfonylureas—the second most widely used antihyperglycemic class after metformin—are lacking. A better understanding of the comparative effectiveness of SGLT2 inhibitors vs sulfonylureas associated with all-cause mortality (a terminal outcome that encompasses the breadth of potential SGLT2 inhibitor benefits) might guide a more informed choice of antihyperglycemic therapy in people with type 2 diabetes.

Sulfonylureas and SGLT2 inhibitors are often used after metformin as second-line antihyperglycemic agents. Given the knowledge gained from randomized clinical trials and the totality of real-world evidence, we hypothesized that, among individuals using metformin and compared with sulfonylureas, SGLT2 inhibitors may be associated with reduced risk of all-cause mortality. In this work, we used the US Department of Veterans Affairs electronic health care databases to evaluate the comparative effectiveness of SGLT2 inhibitors vs sulfonylureas associated the risk of all-cause mortality in persons receiving metformin therapy.

## Methods

### Study Design

Individuals were eligible for the study if they were using metformin therapy between October 1, 2016, and February 29, 2020 (N = 1 025 731). Among these, 397 365 individuals received SGLT2 inhibitors or sulfonylureas within 90 days after use of metformin, with the date of the first SGLT2 inhibitor or sulfonylurea prescription defined as the date of treatment initiation. Persons with a prescription record of SGLT2 inhibitors or sulfonylureas within the past year before treatment initiation did not meet the eligibility criteria (n = 197 470: SGLT2 inhibitors, 34 498; sulfonylureas, 162 972). Individuals would not be further selected if they had been enrolled in the Veterans Affairs Health Care System for less than a year at treatment initiation (n = 156 466: SGLT2 inhibitors, 29 585; sulfonylureas, 126 881) or had a history of type 1 diabetes, estimated glomerular filtration rate (eGFR) less than 30 mL/min/1.73 m^2^, dialysis, or kidney transplant in the year before treatment initiation (n = 143 821: SGLT2 inhibitors, 26 863; sulfonylureas, 116 958). Individuals were then selected on the basis of having measured hemoglobin A_1c_ levels, height, weight, blood pressure, eGFR, and low-density lipoprotein cholesterol levels within the year before treatment initiation, yielding an analytic cohort of 128 293 individuals (SGLT2 inhibitors, 23 870; sulfonylureas, 104 423) (eFigure 1 in the Supplement). Participants were followed up until the occurrence of death or administrative end of follow-up (January 31, 2021). The study was approved by the institutional review board of the Department of Veterans Affairs St Louis Health Care System, St Louis, Missouri, with a waiver of informed consent because of the retrospective nature of the study. This study followed the International Society for Pharmacoeconomics and Outcomes Research (ISPOR) reporting guideline for comparative effectiveness studies.

We used Department of Veterans Affairs Corporate Data Warehouse (CDW) as the data source of this study.^23,24,25,26,27^ The CDW outpatient and inpatient encounters domains were used to collect *International Statistical Classification of Diseases and Related Health Problems, Tenth Revision (ICD-10)* diagnosis codes, *Current Procedural Terminology* codes, and *ICD-10* procedure codes.^27,28,29,30,31,32,33^ Pharmacy data were obtained from the CDW outpatient pharmacy domain and laboratory data were obtained from the CDW laboratory results domain.^34^ The CDW vital signs domain, the CDW patient domain, and VA vital status databases were used to collect demographic information and vital status data.^26,33,35^

### Treatment and Outcome

Use of SGLT2 inhibitors and sulfonylureas was the treatment of the study and was defined based on prescription records. Distribution of medications within the SGLT2 inhibitor and sulfonylurea classes is presented in eTable 1 in the Supplement.

The intention-to-treat effect size, which is the outcome associated with use of an SGLT2 inhibitor or sulfonylurea at treatment initiation, was examined. We also examined the per-protocol effect size, which is the treatment effect size when participants followed a specified treatment protocol for medication use. Two treatment protocols with different clinical implications were specified for per-protocol analyses: continued use of SGLT2 inhibitors or sulfonylureas throughout follow-up and concurrent use of SGLT2 inhibitors and metformin or continued use of SGLT2 inhibitors without metformin throughout follow-up. Discontinued use of a medication was defined based on no record of a prescription refill within 90 days after the end of the supply. Time until all-cause mortality was the outcome of the study.

### Covariates

Covariates that may be different across the 2 arms in observed data were ascertained in the year before treatment initiation. Variables with known associations with treatment selection were used as predefined covariates.^11,12^ Predefined covariates included age, race (White, Black, and other), sex, hemoglobin A_1c_ level, eGFR, systolic blood pressure, diastolic blood pressure, low-density lipoprotein cholesterol level, and body mass index (BMI) (calculated as weight in kilograms divided by height in meters squared). Diseases that may have influenced the choice of treatment, such as congestive heart failure, cardiovascular diseases, cancer, alcoholism, hypoglycemia, diabetic ketoacidosis, acute kidney injury, bladder and urinary tract infections, venous thromboembolism, pancreatitis, bone fracture, and albuminuria (no albuminuria: albumin to creatinine ratio [ACR] ≤30 mg/g, microalbuminuria: ACR >30 to ≤300 mg/g, and macroalbuminuria: ACR >300 mg/g) were also included as predefined covariates.^36^ Prescription of glucagonlike peptide-1 receptor agonists, dipeptidyl peptidase-4 inhibitors, thiazolidinediones, insulin, α-glucosidase inhibitors, meglitinides, amylin analogues, statins, angiotensin-converting enzyme inhibitors, angiotensin receptor blockers, β-blockers, diuretics, and calcium channel blockers were also included as predefined covariates.^36^ Smoking status (never, former, current), type of health care system in which the antihyperglycemic was prescribed at treatment initiation (hospital system or outpatient clinic), and the calendar year of treatment initiation were also included as predefined covariates. To account for potential nonlinear associations between continuous variables and treatment assignment, all continuous variables were transformed into restricted cubic splines unless otherwise specified.

High-dimensional covariate data from 7 data domains, including outpatient *ICD-10* diagnostic codes, outpatient *Current Procedural Terminology* codes, inpatient *ICD-10* diagnostic codes, inpatient *Current Procedural Terminology* codes, and inpatient *ICD-10* procedure codes for surgeries, pharmacy records, and laboratory results, were additionally used to further reduce potential biases.^37,38^ Participants’ health records within 1 year before treatment initiation were used to construct the high-dimensional propensity score. First, the top 300 frequently occurring items (eg, diagnosis, procedure, laboratory test result) among participants from each of the 7 data domains were individually categorized into 3 binary variables: ever occurred (occurred more than once in the participant), sometimes occurred (occurred more than in 50% of other participants), and frequently occurred (occurred more than in 75% of other participants). Univariate associations between each variable with treatment assignment were evaluated based on relative risk, and the 300 variables with the largest relative risks were selected for constructing the high-dimensional propensity score. Selections were conducted independently in the overall cohort and within each subgroup.

To estimate the per-protocol effect size, both predefined and high-dimensional covariates were time updated. High-dimensional variables for per-protocol analyses were selected based on their association with adherence to the treatment protocol.^39^

### Statistical Analysis

Characteristics of the SGLT2 inhibitor and sulfonylurea arms are described as mean (SD) or number (percentage). The overall analytic approach flowchart is presented in eFigure 2 in the Supplement. We used overlap weighting based on high-dimensional propensity score (using predefined and algorithmically selected high-dimensional covariates) to balance the exposure groups (SGLT2 inhibitors and sulfonylureas).^40,41^ The high-dimensional propensity score was estimated from logistic regression, with both predefined covariates and algorithmically selected high-dimensional variables used to predict treatment assignment. We then applied overlap weighting to the cohort to account for the different baseline characteristics between patients in the real-world setting using SGLT2 inhibitors and sulfonylureas. The weighting was constructed as the probability of receiving the opposite treatment (1 minus the probability of receiving the assigned treatment). The overlap weight for each participant with possible minimal value of 0 and maximum value of 1, without stabilization or trimming, was used.^40^ To assess the success of balancing, we evaluated the propensity score distributions and covariate standardized mean differences before and after adjustment (eFigure 3 and eFigure 4 in the Supplement).

To estimate the risk between initiation of SGLT2 inhibitors and sulfonylureas on all-cause mortality, a Cox proportional hazards model with the overlap weighting was applied. The mortality rate per 1000 person-years in individuals initiating SGLT2 inhibitors and those initiating sulfonylureas and the event rate difference between the 2 groups were computed from the survival probability based on all data collected during the follow-up, with survival probability estimated based on the hazard ratio (HR) and underlying risk generated from Breslow estimator.^42^ Multiple subgroup analyses were conducted in predefined subgroups based on those younger and older than 65 years, baseline cardiovascular disease status, eGFR status (≥90, <90 to ≥60, <60 to ≥45, and <45 mL/min/1.73 m^2^), albuminuria status, BMI categories (≤25, 25-≤30, and >30), and use of medications, including insulin, angiotensin-converting enzyme inhibitors, angiotensin receptor blockers, and diuretics. High-dimensional propensity scores and weights for each subgroup were constructed independently.

In addition to the intention-to-treat effect size, we examined the per-protocol effect sizes of SGLT2 inhibitors and sulfonylureas, based on participants’ adherence to the defined treatment protocol.^43,44,45^ The per-protocol effect sizes were estimated by inverse weighing the probability of nonadherence to the protocol at every time point.^45,46,47^ We first estimated the probability of adherence at each time point *k* within participants who were adherent to the treatment protocol at the previous time point (*k*-1). The probability was estimated based on time updated covariates. The inverse probability of adherence weighting at time *t* was then constructed as 
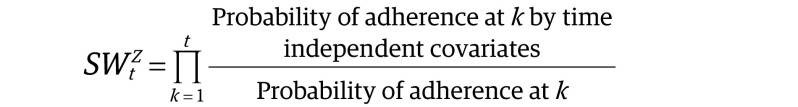
where *Z* is an indicator of adherence and the stabilized factor in the numerator was the probability of adherence based on time-independent covariates, including age, race, sex, type of health care system, and year of treatment initiation. The stabilized adherence weights were multiplied with treatment weights to balance baseline covariates (done using overlap weighting) and generate summarized weights. The summarized weights were further truncated at both tails to reduce the bias and variance. Weights were applied to pooled logistic regression to estimate the per-protocol effect size, with follow-up time treated as a restricted cubic spline and knots placed at 180, 360, 540, 720, and 900 days.

The robustness of our result was examined through multiple sensitivity analyses. We (1) censored follow-up at February 29, 2020, to remove the influence of COVID-19 on the outcome through altered care of patients with diabetes, risk of death due to COVID-19, and other factors related to COVID-19; (2) applied the inverse probability of treatment weighting to balance characteristics between SGLT2 inhibitors and sulfonylureas as an alternative to the overlap weighting; (3) in consideration of the potential correlation between high-dimensional selected variables, applied least absolute shrinkage and selection operator regression to estimate the propensity score; (4) examined the association in 2 enrollment periods (2016, 2017) and separately (2018, 2019, 2020) because antihyperglycemic prescribing preferences may have changed over time; and (5) removed mortality happening in the first 180 days of follow up, and separately, removed mortality happening in the first 90 days of follow-up, because these events were most likely not related to the treatments.

To detect the presence of spurious biases, we followed the approach outlined by Lipsitch and colleagues^48^ to examine the association between SGLT2 inhibitors and chronic lower respiratory disease as a negative outcome control. To our knowledge, there is no evidence suggesting a causal association exists; therefore, we would expect a priori that a successful application of this negative outcome control test would yield a null association. Similarly, we examined the association between SGLT2 inhibitors and BMI decrease by greater than 10%, and separately, BMI increase by greater than 10% as positive outcome controls. We would expect to observe that SGLT2 inhibitors was associated with an increased risk of a BMI decrease and a reduced risk of a BMI increase, based on established knowledge from randomized clinical trials and real-world evidence.

Based on 500 times bootstrapping, 95% CIs were generated for rate and rate difference. A 95% CI of a ratio that does not cross 1 or of a rate that does not cross 0 was considered statistically significant. All analyses were done using SAS Enterprise Guide, version 7.1 (SAS Institute Inc).

## Results

The cohort included 128 293 participants: 23 870 individuals with new use of SGLT2 inhibitors and 104 423 individuals with new use of sulfonylureas. Mean (SD) age was 64.60 (9.84) years; 122 096 men (95.17%) and 6197 women (4.83%) were included. The demographic and health characteristics in the overall cohort and by treatment arm before adjustment are provided in eTable 2 in the Supplement; characteristics after adjustment are reported in Table 1.

**Table 1.** Demographic and Health Characteristics After Adjustment

Baseline characteristics	No. (%)			Absolute standardized difference^a^
	Overall cohort	SGLT2 inhibitor	Sulfonylurea	
No.	128 293	23 870 (18.61)	104 423 (81.39)	
Age, mean (SD), y	64.60 (9.84)	64.60 (9.81)	64.60 (9.87)	<0.01
Race				
White	97 772 (76.21)	18 191 (76.21)	79 581 (76.21)	<0.01
Black	23 927 (18.65)	4452 (18.65)	19 475 (18.65)	<0.01
Other	6594 (5.14)	1227 (5.14)	5367 (5.14)	<0.01
Sex				
Male	122 096 (95.17)	22 717 (95.17)	99 379 (95.17)	<0.01
Female	6197 (4.83)	1153 (4.83)	5044 (4.83)	
eGFR, mean (SD), mL/min/1.73 m^2^	79.07 (18.91)	79.07 (18.84)	79.07 (18.99)	<0.01
eGFR status, mL/min/1.73 m^2^				
≥90	37 806 (29.47)	7168 (30.03)	30 638 (29.34)	0.02
≥60 to <90	68 771 (53.60)	12 403 (51.96)	56 368 (53.98)	0.04
≥45 to >60	17 383 (13.55)	3745 (15.69)	13 638 (13.06)	0.08
30 to >45	4334 (3.38)	554 (2.32)	3780 (3.62)	0.08
HbA_1c_, mean (SD), %	8.60 (1.59)	8.60 (1.60)	8.60 (1.59)	<0.01
BMI, mean (SD)	33.79 (6.62)	33.79 (6.67)	33.79 (6.56)	<0.01
Low-density lipoprotein cholesterol, mean (SD), mg/dL	83.13 (38.22)	83.13 (36.83)	83.13 (39.56)	<0.01
Blood pressure, mean (SD), mm Hg				
Systolic	132.20 (16.60)	132.20 (16.61)	132.20 (16.60)	<0.01
Diastolic	75.87 (10.13)	75.87 (10.10)	75.87 (10.15)	<0.01
Congestive heart failure	11 226 (8.75)	2089 (8.75)	9137 (8.75)	<0.01
Alcoholism	6928 (5.4)	1289 (5.4)	5639 (5.4)	<0.01
Bone fracture	1424 (1.11)	265 (1.11)	1159 (1.11)	<0.01
Cancer	25 248 (19.68)	4698 (19.68)	20 550 (19.68)	<0.01
Cardiovascular disease	44 197 (34.45)	8223 (34.45)	35 974 (34.45)	<0.01
Diabetic ketoacidosis	436 (0.34)	81 (0.34)	355 (0.34)	<0.01
Hypoglycemia	2899 (2.26)	539 (2.26)	2360 (2.26)	<0.01
Pancreatitis	1578 (1.23)	294 (1.23)	1284 (1.23)	<0.01
Bladder and urinary tract infection	2758 (2.15)	513 (2.15)	2245 (2.15)	<0.01
Venous thromboembolism	808 (0.63)	150 (0.63)	658 (0.63)	<0.01
Acute kidney injury	10 854 (8.46)	2019 (8.46)	8834 (8.46)	<0.01
Albuminuria^b^				
No albuminuria	74 808 (58.31)	13 919 (58.31)	60 889 (58.31)	<0.01
Microalbuminuria	44 633 (34.79)	8304 (34.79)	36 329 (34.79)	<0.01
Macroalbuminuria	8865 (6.91)	1649 (6.91)	7216 (6.91)	<0.01
Insulin	61 465 (47.91)	11 436 (47.91)	50 029 (47.91)	<0.01
DPP4	19 116 (14.9)	3557 (14.9)	15 559 (14.9)	<0.01
GLP1	7929 (6.18)	1475 (6.18)	6453 (6.18)	<0.01
Thiazolidinedione	4760 (3.71)	886 (3.71)	3874 (3.71)	<0.01
ACE inhibitor/ARB	82 954 (64.66)	15 434 (64.66)	67 520 (64.66)	<0.01
Calcium channel blocker	36 615 (28.54)	6812 (28.54)	29 802 (28.54)	<0.01
β-Blocker	59 720 (46.55)	11 111 (46.55)	48 609 (46.55)	<0.01
Diuretic	52 562 (40.97)	9780 (40.97)	42 782 (40.97)	<0.01
Statin	102 750 (80.09)	19 117 (80.09)	83 632 (80.09)	<0.01
Type of health care system				
Outpatient clinic	73 653 (57.41)	13 704 (57.41)	59 949 (57.41)	<0.01
Hospital system	54 640 (42.59)	10 166 (42.59)	44 474 (42.59)	
Year of treatment initiation				
2016	2989 (2.33)	556 (2.33)	2433 (2.33)	<0.01
2017	24 222 (18.88)	4507 (18.88)	19 715 (18.88)	<0.01
2018	36 717 (28.62)	6832 (28.62)	29 886 (28.62)	<0.01
2019	56 051 (43.69)	10 429 (43.69)	45 622 (43.69)	<0.01
2020	8313 (6.48)	1547 (6.48)	6767 (6.48)	<0.01
Smoking status				
Never	70 189 (54.71)	13 059 (54.71)	57 130 (54.71)	<0.01
Former	32 214 (25.11)	5994 (25.11)	26 221 (25.11)	<0.01
Current	25 800 (20.11)	4800 (20.11)	20 999 (20.11)	<0.01
Follow-up, mean (SD), y	2.20 (0.91)	2.17 (0.90)	2.22 (0.91)	0.05

Abbreviations: ACE inhibitor/ARB, angiotensin-converting enzyme inhibitor/angiotensin-receptor blocker; BMI, body mass index (calculated as weight in kilograms divided by height in meters squared); DPP4, dipeptidyl peptidase-4 inhibitor; eGFR, estimated glomerular filtration rate; GLP1, glucagonlike peptide-1; HbA_1c_, glycated hemoglobin; SGLT2, sodium-glucose cotransporter 2.

SI conversion factor: To convert low-density lipoprotein cholesterol to millimoles per liter, multiply by 0.0259.

^a^ Standardized difference less than 0.1 indicates good balance between 2 groups.

^b^ Albuminuria status categorized as no albuminuria (defined as albumin to creatinine ratio [ACR]<30 mg/g), microalbuminuria (ACR 30 to <300 mg/g), and macroalbuminuria (ACR≥300 mg/g).

**Figure 1.**
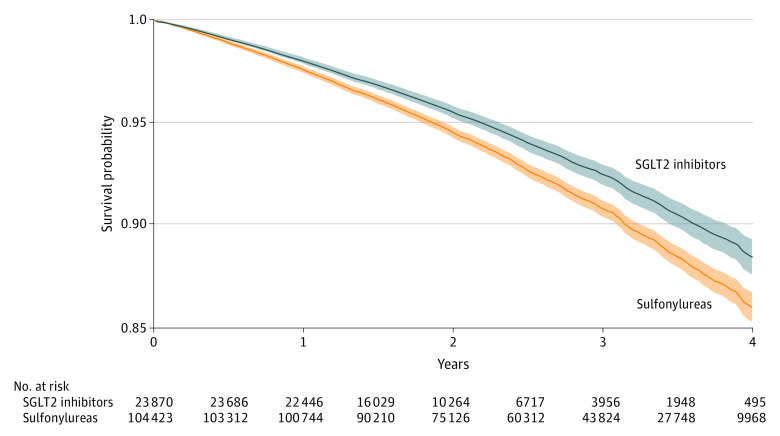
Adjusted Intention-to-Treat Survival Probability for All-Cause Mortality Survival probability in the sodium-glucose cotransporter 2 (SGLT2) inhibitor and sulfonylurea treatment arms. Shaded bands represent 95% CIs.

**Figure 2.**
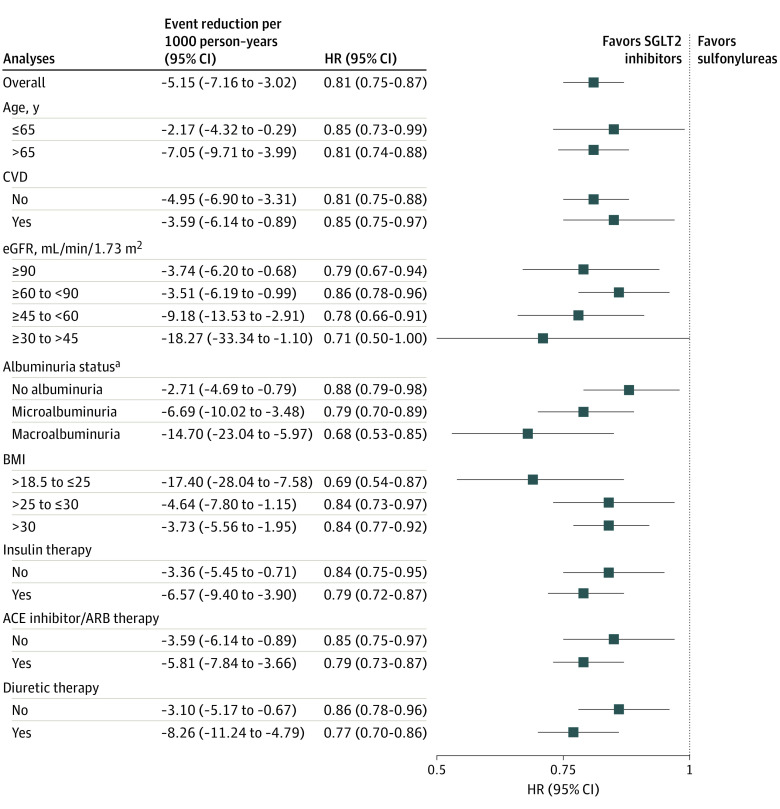
Intention-to-Treat Hazard Ratios (HRs) and Event Rate Reduction for All-Cause Mortality in the Overall Cohort and Prespecified Subgroups Hazard ratios of all-cause mortality in the overall cohort and by age category, cardiovascular disease (CVD) status, estimated glomerular filtration (eGFR) category, albuminuria category, body mass index (BMI) category (calculated as weight in kilograms divided by height in meters squared), and baseline use of metformin, insulin, angiotensin-converting enzyme inhibitors or angiotensin receptor blockers (ACE inhibitor/ARB), and diuretics. SGLT2 indicates sodium-glucose cotransporter-2. ^a^Albuminuria status was categorized using albumin to creatinine ratio (ACR): no albuminuria (ACR ≤30 mg/g), microalbuminuria (ACR >30 to ≤300 mg/g), and macroalbuminuria (ACR >300 mg/g).

Adjusted survival probability for all-cause mortality and number of participants at risk during the follow-up period are provided in Figure 1. Compared with new use of sulfonylureas, new use of SGLT2 inhibitors was associated with a reduced risk of all-cause mortality (HR, 0.81; 95% CI, 0.75-0.87). Adjusted event rate differences suggested that, compared with sulfonylureas, use of SGLT2 inhibitors was associated with −5.15 (95% CI, −7.16 to −3.02) deaths per 1000 person-years. In prespecified subgroup analyses, SGLT2 inhibitor use was associated with a reduced risk of all-cause mortality, regardless of age, cardiovascular disease status, eGFR category, albuminuria status, BMI category, and baseline use of insulin, angiotensin-converting enzyme inhibitors, angiotensin receptor blockers, and diuretics (Figure 2; eTable 3 in the Supplement). Estimates of absolute rate differences showed that event rate reduction was higher in participants with cardiovascular disease, lower eGFR category, and albuminuria (microalbuminuria or macroalbuminuria) (Figure 2; eTable 3 in the Supplement).

**Figure 3.**
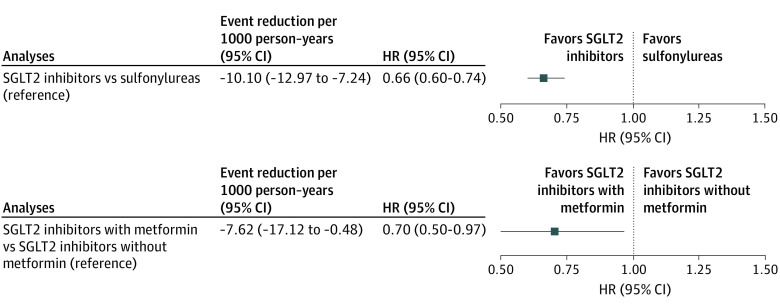
Per-Protocol Hazard Ratios (HRs) and Event Rate Reduction for All-Cause Mortality Hazard ratios of all-cause mortality in continued use of sodium-glucose cotransporter 2 (SGLT2) inhibitors or sulfonylureas (reference group) throughout follow-up (top graph) and continued use of SGLT2 inhibitors with metformin or SGLT2 inhibitors without metformin (reference group) throughout follow up (bottom graph).

In a prespecified protocol that required continued use of SGLT2 inhibitors or sulfonylureas throughout the study duration, SGLT2 inhibitor use was associated with a reduced risk of all-cause mortality compared with sulfonylureas (HR, 0.66; 95% CI, 0.60-0.74; event rate difference, −10.10; 95% CI, −12.97 to −7.24 deaths per 1000 person-years) (Figure 3; eTable 4 in the Supplement).

In another prespecified protocol (applied to the SGLT2 inhibitor arm), which required continued use of SGLT2 inhibitors with metformin or SGLT2 inhibitors without metformin throughout the study duration, compared with SGLT2 inhibitor use without metformin, SGLT2 inhibitor use with metformin was associated with a reduced risk of all-cause mortality (HR, 0.70; 95% CI, 0.50-0.97; event rate difference, −7.62; 95% CI, −17.12 to −0.48 deaths per 1000 person-years) (Figure 3, eTable 5 in the Supplement).

We conducted several sensitivity analyses to test the robustness of study results (Table 2). First, because the COVID-19 pandemic may have altered care of patients with diabetes and these patients may have a higher risk of death due to COVID-19, and to eliminate bias that may be introduced by these and other factors related to COVID-19, we censored participants on February 29, 2020 (before the onset of the pandemic in the US); the results show that the SGLT2 inhibitor arm exhibited less risk of all-cause mortality compared with the sulfonylurea arm. Second, as an alternative to the overlap-weighting method used in our primary approach, we used the inverse probability treatment-weighting method to balance characteristics of the 2 exposure groups; the result was consistent in that the magnitude and direction of point estimates were consistent with those in the primary analyses. Third, least absolute shrinkage and selection operator regression was used to account for potential increased correlation between high-dimensional variables; the result was consistent with the primary analysis. Fourth, to examine whether the observed association of SGLT2 inhibitors and all-cause mortality varied depending on temporal differences in the availability of the antihyperglycemic medication and prescription criteria, we examined the association of SGLT2 inhibitors and the outcome in 2016 and 2017 when SGLT2 inhibitor agents were less accessible and in 2018 and 2019 when SGLT2 inhibitor use became relatively more popular. Compared with the sulfonylurea arm, SGLT2 inhibitor use was associated with a reduced risk of all-cause mortality in the 2 periods examined (HR, 0.88; 95% CI, 0.77-0.99 in 2016 and 2017; HR, 0.77; 95% CI, 0.70-0.85 in 2018, 2019, and 2020). Fifth, we conducted analyses in which we removed individuals with an event occurring in the first 180 days of follow-up, because it is unlikely that these events are related to exposure to the antihyperglycemic agent; results showed that, compared with sulfonylureas, SGLT2 inhibitor use was associated with a reduced risk of all-cause mortality (HR, 0.82; 95% CI, 0.76-0.88). These results were also consistent in analyses that removed individuals with an event occurring in the first 90 days of follow-up (HR, 0.84; 95% CI, 0.78-0.91).

**Table 2.** Sensitivity Analyses for Comparison of SGLT2 Inhibitors and Sulfonylureas as Reference Group on Risk of All-Cause Mortality

Sensitivity analyses	Hazard ratio	Death rate per 1000 person-years (adjusted 95% CI)		Event reduction per 1000 person-years (95% CI)
		SGLT2 inhibitors	Sulfonylureas	
Censored on February 29, 2020	0.82 (0.74 to 0.91)	20.84 (18.85 to 22.72)	25.11 (23.85 to 26.77)	−4.47 (−6.63 to −2.42)
Inverse probability treatment weight	0.87 (0.77 to 0.99)	22.19 (19.65 to 24.89)	25.49 (24.82 to 26.23)	−3.32 (−5.97 to −0.32)
Propensity score–based on LASSO regression	0.79 (0.74 to 0.85)	22.86 (21.32 to 24.60)	28.69 (27.49 to 29.77)	−5.78 (−7.89 to −3.56)
Within patients enrolled in 2016 and 2017	0.88 (0.77 to 0.99)	25.20 (22.22 to 28.22)	28.33 (26.40 to 30.37)	−3.12 (−3.08 to −0.21)
Within patients enrolled in 2018, 2019, and 2020	0.77 (0.70 to 0.85)	21.44 (19.46 to 23.13)	27.36 (25.91 to 29.07)	−5.82 (−8.07 to −3.96)
Excluded patients with events within 180 d from treatment initiation^a^	0.82 (0.76 to 0.88)	20.80 (19.43 to 22.25)	24.68 (23.74 to 25.86)	−3.94 (−5.49 to −2.31)
Excluded patients with events within 90 d from treatment initiation^b^	0.84 (0.78 to 0.91)	22.05 (20.60 to 23.49)	26.69 (25.67 to 27.81)	−4.71 (−6.40 to −2.73)

Abbreviations: LASSO, least absolute shrinkage and selection operator; SGLT2, sodium-glucose cotransporter 2.

^a^ A total of 181 (18.81%) of the events from the SGLT2 inhibitor group and 1098 (14.04%) of the events from the sulfonylurea group were excluded.

^b^ A total of 85 (9.96%) of the events from the SGLT2 inhibitor group and 502 (6.42%) of the events from the sulfonylurea group were excluded.

To test for possible spurious biases, we examined the association between SGLT2 inhibitors and the risk of chronic lower respiratory disease as a negative outcome control, with no prior knowledge suggesting a causal association. Our analyses suggested there was no significant association between SGLT2 inhibitors and chronic lower respiratory disease (HR, 1.04; 95% CI, 0.97-1.13) (eFigure 5a in the Supplement).

To test whether our approach would reproduce a priori knowledge, we examined the association between SGLT2 inhibitors and sulfonylureas and the risk of more than a 10% increase and, separately, more than a 10% decrease in BMI as positive outcome controls. Established knowledge from randomized clinical trials and real-world evidence suggests that SGLT2 inhibitor use is associated with a reduction in BMI, whereas use of sulfonylureas is associated with an increase in BMI. Our results suggest that, compared with sulfonylureas, SGLT2 inhibitor use was associated with an increased risk of a more than 10% decrease in BMI (HR, 1.44; 95% CI, 1.38-1.50) and decreased risk of a more than 10% increase in BMI (HR, 0.52; 0.48-0.56) (eFigure 5b and 5c in the Supplement).

## Discussion

In this work, we leveraged the breadth and depth of the electronic health care databases of the US Department of Veterans Affairs and methodologic advances to evaluate the comparative effectiveness of SGLT2 inhibitors vs sulfonylureas associated with the risk of all-cause mortality among individuals using metformin for treatment of type 2 diabetes. The results suggest that, compared with sulfonylureas, SGLT2 inhibitors use was associated with a reduced risk of all-cause mortality. The association was evident in individuals with and without cardiovascular disease, regardless of eGFR category and albuminuria status, and in several other prespecified subgroups. Per-protocol analyses showed that combined use of SGLT2 inhibitors and metformin was associated with a reduced risk for all-cause mortality compared with SGLT2 inhibitors alone. The results were robust to challenge in multiple sensitivity analyses. The testing of negative and positive outcome controls yielded results consistent with a priori expectations.

Sulfonylureas are the most commonly used second-line antihyperglycemic medications—accounting for 37% of the global market share of antihyperglycemics.^49,50^ Despite this, evidence on the comparative effectiveness of sulfonylureas vs SGLT2 inhibitors, the newest class of second-line antihyperglycemics, is lacking. Our results provide data suggesting that, among metformin users, compared with new use of sulfonylureas, new use of SGLT2 inhibitors was associated with a reduced risk of all-cause mortality in the overall cohort and in several prespecified subgroups. Use of SGLT2 inhibitors was associated with a reduced risk of all-cause mortality in individuals with and without cardiovascular disease, regardless of eGFR category and albuminuria status. Juxtaposed against the background that nearly all randomized clinical trials of SGLT2 inhibitors enrolled high-risk groups (patients with or at high risk for cardiovascular disease and those with kidney disease or albuminuria), our results complement evidence from randomized clinical trials and suggest that the salutary association between SGLT2 inhibitors and the risk of mortality likely extends to lower risk groups, including those without cardiovascular disease, with eGFR greater than 60 mL/min/1.73 m^2^, and with no albuminuria or microalbuminuria. Our findings also suggest that, although estimates of relative risk (HRs) were consistently reduced across all subgroups, estimates of the absolute rate reduction suggested that those in the lower eGFR categories, and microalbuminuria and macroalbuminuria categories may derive the highest absolute risk reduction owing to a higher baseline risk in these subgroups.

A consensus report by the American Diabetes Association and the European Association for the Study of Diabetes recommends metformin as the preferred initial antihyperglycemic for most people with type 2 diabetes and suggests that stepwise addition of medication to decrease glucose levels is preferred to initial combination therapy.^51,52^ However, increasingly, some clinical practice guidelines are relaxing these recommendations. Guidelines from the European Society of Cardiology suggest initiation of SGLT2 inhibitors in patients with type 2 diabetes who are at high or very high cardiovascular risk irrespective of whether they are treatment naive or already receiving metformin.^53^ The newly released KDIGO guidelines suggest that metformin and SGLT2 inhibitors should be considered as first-line treatment in patients with type 2 diabetes and kidney disease.^54^ Increasingly, second-line antihyperglycemics are often initiated without first-line metformin therapy.^55^ In the SGLT2 inhibitors trials involving patients with type 2 diabetes, baseline metformin use ranged from 58% to 82%.^56^ In this study, we designed a per-protocol analysis to gain a better understanding whether continued metformin use with the addition of SGLT2 inhibitors was contributing to risk reduction. The results suggest that combined use of SGLT2 inhibitors with metformin was associated with a reduced risk of mortality compared with SGLT2 inhibitors alone. Our results suggest that metformin contributes to risk reduction and inform the discussion around the role of metformin in this new era of antihyperglycemics. Owing to substantial cost difference vs SGLT2 inhibitors, long-standing safety record, tolerability, and beneficial metabolic profile, metformin may still be the preferred choice as a first-line antihyperglycemic agent in type 2 diabetes. A head-to-head evaluation of SGLT2 inhibitor vs metformin therapy will be needed before fully endorsing status of SGLT2 inhibitors as a first-line antihyperglycemic agent.

In considering initiation of second-line antihyperglycemics, cost is often a major factor that influences choice of an agent. A recent analysis estimated that, among Medicare Part D plans in 2019, the total annual and out-of-pocket cost for sulfonylureas was $31, the total annual cost for SGLT2 inhibitors ranged from $5967 to $6118, and annual out-of-pocket-cost for SGLT2 inhibitors ranged from $1298 to $1615.^57^ However, despite higher treatment cost and owing to their salutary properties in reducing complication costs and gains in quality-adjusted life-years, SGLT2 inhibitors are now considered to be cost-saving or cost-effective.^58^ Nevertheless, the substantially higher out-of-pocket cost of SGLT2 inhibitors limits access to many patients who may benefit from these newer agents and might contribute to inequities. Policy measures aimed at reducing out-of-pocket costs and facilitating access will be important to mitigate to the extent possible potential financial contributors to health inequities.

The mechanisms underpinning the association between SGLT2 inhibitors and risk of death are not entirely clear. Experimental and clinical evidence suggest several putative mechanisms that might explain the beneficial properties of SGLT2 inhibitors on the risk of death, including hemodynamic (eg, natriuresis and osmotic diuresis, blood pressure reduction), metabolic (eg, weight loss), reduced inflammation and oxidative stress, and improved vascular endothelial function.^59,60^ It is plausible that several of these putative mechanistic pathways are contributing to the observed association of SGLT2 inhibitors with risk of all-cause mortality.

### Strengths and Limitations

The study has several strengths. We developed our research aim, study design, and execution to specifically address a knowledge gap of the comparative effectiveness of incident use of SGLT2 inhibitors vs sulfonylureas on the risk of all-cause mortality among people receiving metformin. To our knowledge, this issue has not been and is unlikely to be addressed in randomized clinical trials; at this time, there are no registered clinical trials (finished or ongoing) addressing the comparative effectiveness of SGLT2 inhibitors vs sulfonylureas. We used large-scale real-world data from the US Department of Veterans Affairs, which operates the largest integrated health care system in the US; Veterans Affairs data are captured during routine clinical care, which might more closely recapitulate real-world experiences. Although there is a substantial cost differential between SGLT2 inhibitors and sulfonylureas, initiation or discontinuation of these agents is less likely to be influenced by financial considerations in the Veterans Affairs system (a US government–funded health care system that provides comprehensive health care benefits, including prescription drug coverage, to discharged veterans of the US armed forces). We used a design to examine individuals initiating therapy, applied advanced statistical methodologies, including overlap weighting and high-dimensional variable selection algorithms, and reported both an intention-to-treat effect size, which estimates effectiveness of SGLT2 inhibitors at the level of observed adherence in our cohort, and per-protocol analysis, which accounts for nonadherence and offers estimates of effectiveness that may be more generalizable across different settings.^45^ In addition, we specified our per-protocol analyses to address questions relevant to the clinical community, in particular, evaluation of the comparative effectiveness of SGLT2 inhibitors with and without metformin. We examined the comparative effectiveness in prespecified subgroups of interest to the clinical community. In addition to reporting relative risk, we reported absolute rate differences in the overall cohort and in prespecified subgroups with different baseline risks that may be clinically meaningful in informing the choice of antihyperglycemic medications. We tested the robustness of the results in multiple sensitivity analyses, applied a negative control to detect spurious associations,^48^ and applied positive controls to test whether our approach would reproduce established a priori knowledge.

This study has several limitations. We relied on observational real-world data from the US Department of Veterans Affairs to build a cohort that mostly comprised older, White, and male participants, which may limit the generalizability of study findings. We note the substantial difference in baseline characteristics between the SGLT2 inhibitor and sulfonylurea groups (individuals using SGLT2 inhibitors were older and had a higher burden of several comorbidities, including cardiovascular and kidney disease). Although our analytic approach evaluated SGLT2 inhibitors vs sulfonylureas (an active comparator), considered known confounders, and applied a high-dimensional variable selection algorithm to more comprehensively capture potential confounding, we cannot rule out the possibility of residual confounding. We estimated the intention-to-treat effect sizes, which may be limited by variable nonadherence among study participants; however, we also evaluated the study question in prespecified per-protocol analyses that accounted for nonadherence. We did not examine differences within the SGLT2 inhibitors and sulfonylureas and did not examine the risk of adverse events.

## Conclusions

The results of this study suggest that, compared with sulfonylureas, SGLT2 inhibitor use was associated with reduced risk of all-cause mortality among individuals using metformin for treatment of type 2 diabetes. The association was evident in those with and without cardiovascular disease, regardless of eGFR category and albuminuria status, and in several other prespecified subgroups. Per-protocol analyses suggested that combined use of SGLT2 inhibitors and metformin was associated with reduced risk of all-cause mortality compared with SGLT2 inhibitors alone. The results provide real-world evidence on the association of SGLT2 inhibitor use with the risk of all-cause death; the results may help guide the choice of antihyperglycemic therapy in people with type 2 diabetes.

## Notes

Supplement. **eFigure 1.** Cohort Flow **eFigure 2.** Overall Analytic Approach Flowchart **eFigure 3.** Propensity Score Distribution of the SGLT2i and Sulfonylurea Arms **eFigure 4.** Standardized Differences Between the SGLT2i and Sulfonylurea Arms Before and After Adjustment **eFigure 5.** Adjusted Survival Probability for Negative and Positive Outcome Controls **eTable 1.** Distribution of Medications Within Each Antihyperglycemic Class at Treatment Initiation **eTable 2.** Demographic and Health Characteristics of the Unadjusted Cohort **eTable 3.** Intention-to-Treat Hazard Ratios, Death Rates, and Event Reductions in the Overall Cohort and in Subgroups **eTable 4.** Adherence Rate, Estimated Event Rate, Adjusted Hazard Ratio, and Estimated Event Reduction for All-cause Mortality Based on Per-Protocol Analyses for Continued Use of SGLT2i or Sulfonylureas **eTable 5.** Adherence Rate, Estimated Event Rate, Adjusted Hazard Ratio, and Estimated Event Reduction for All-cause Mortality Based on Per-Protocol Analyses for Continued Use of SGLT2i With and Without Metformin
