# Supplementary material for: Comparative Effectiveness of Sodium-Glucose Cotransporter 2 Inhibitors vs Sulfonylureas in Patients With Type 2 Diabetes
Source: JAMA Intern Med. 2021 Jun 28;181(8):1043–53. doi: 10.1001/jamainternmed.2021.2488 (PMC8240007; doi:10.1001/jamainternmed.2021.2488)
Supplement: Supplement. — eFigure 1. Cohort Flow eFigure 2. Overall Analytic Approach Flowchart eFigure 3. Propensity Score Distribution of the SGLT2i and Sulfonylurea Arms eFigure 4. Standardized Differences Between the SGLT2i and Sulfonylurea Arms Before and After Adjustment eFigure 5. Adjusted Survival Probability for Negative and Positive Outcome Controls eTable 1. Distribution of Medications Within Each Antihyperglycemic Class at Treatment Initiation eTable 2. Demographic and Health Characteristics of the Unadjusted Cohort eTable 3. Intention-to-Treat Hazard Ratios, Death Rates, and Event Reductions in the Overall Cohort and in Subgroups eTable 4. Adherence Rate, Estimated Event Rate, Adjusted Hazard Ratio, and Estimated Event Reduction for All-cause Mortality Based on Per-Protocol Analyses for Continued Use of SGLT2i or Sulfonylureas eTable 5. Adherence Rate, Estimated Event Rate, Adjusted Hazard Ratio, and Estimated Event Reduction for All-cause Mortality Based on Per-Protocol Analyses for Continued Use of SGLT2i With and Without Metformin [file jamainternmed-e212488-s001.pdf]

Supplementary Online Content

Xie Y, Bowe B, Gibson AK, McGill JB, Maddukuri G, Al-Aly Z. Comparative effectiveness of sodium-glucose cotransporter 2 inhibitors vs sulfonylureas in patients with type 2 diabetes. *JAMA Intern Med*. Published online June 28, 2021. doi:10.1001/jamainternmed.2021.2488

eFigure 1. Cohort Flow

eFigure 2. Overall Analytic Approach Flowchart

eFigure 3. Propensity Score Distribution of the SGLT2i and Sulfonylurea Arms

eFigure 4. Standardized Differences Between the SGLT2i and Sulfonylurea Arms Before and After Adjustment

eFigure 5. Adjusted Survival Probability for Negative and Positive Outcome Controls

eTable 1. Distribution of Medications Within Each Antihyperglycemic Class at Treatment Initiation

eTable 2. Demographic and Health Characteristics of the Unadjusted Cohort

eTable 3. Intention-to-Treat Hazard Ratios, Death Rates, and Event Reductions in the Overall Cohort and in Subgroups

eTable 4. Adherence Rate, Estimated Event Rate, Adjusted Hazard Ratio, and Estimated Event Reduction for All-cause Mortality Based on Per-Protocol Analyses for Continued Use of SGLT2i or Sulfonylureas

eTable 5. Adherence Rate, Estimated Event Rate, Adjusted Hazard Ratio, and Estimated Event Reduction for All-cause Mortality Based on Per-Protocol Analyses for Continued Use of SGLT2i With and Without Metformin

This supplementary material has been provided by the authors to give readers additional information about their work.

eFigure 1. Cohort flow

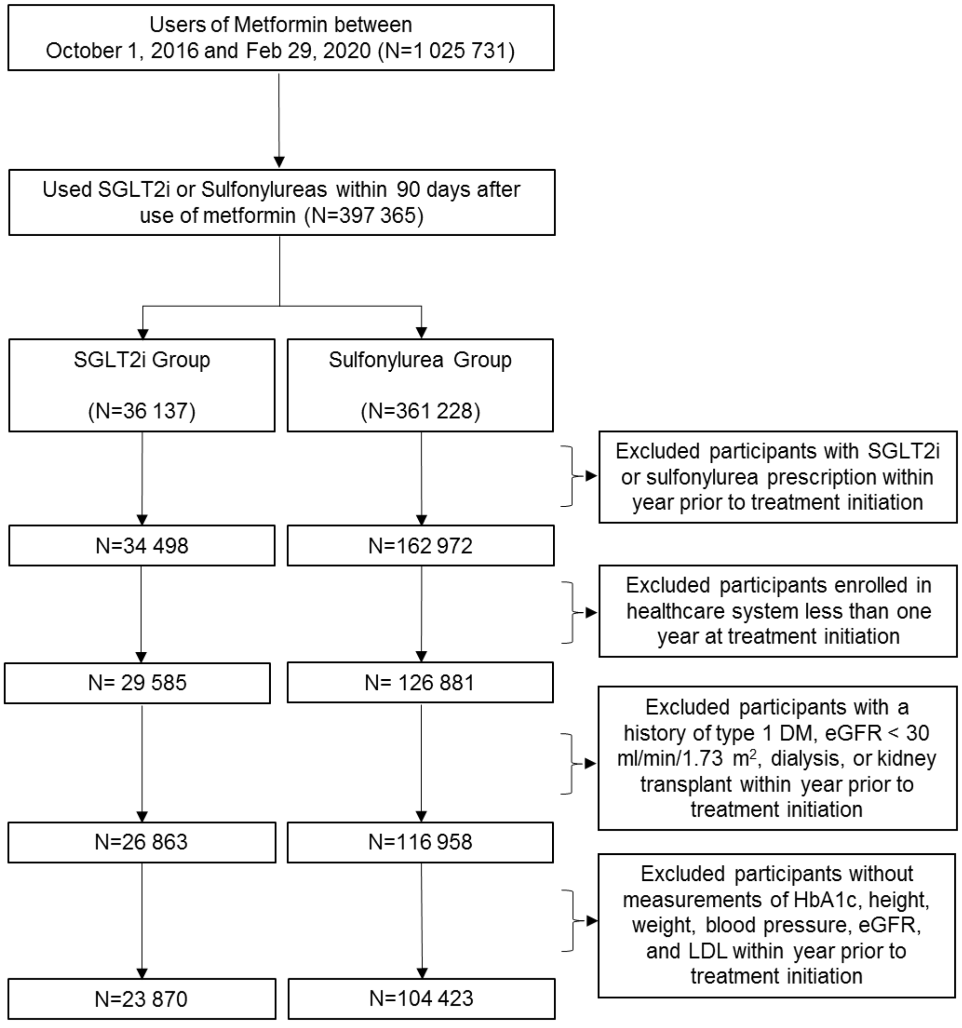

SGLT2i=sodium-glucose co-transporter-2 inhibitor. DM=diabetes mellitus. eGFR= estimated glomerular filtration rate. HbA1c=glycated hemoglobin. LDL=low-density lipoprotein.

© 2021 Xie Y et al. *JAMA Internal Medicine*.

eFigure 2. Overall analytic approach flowchart

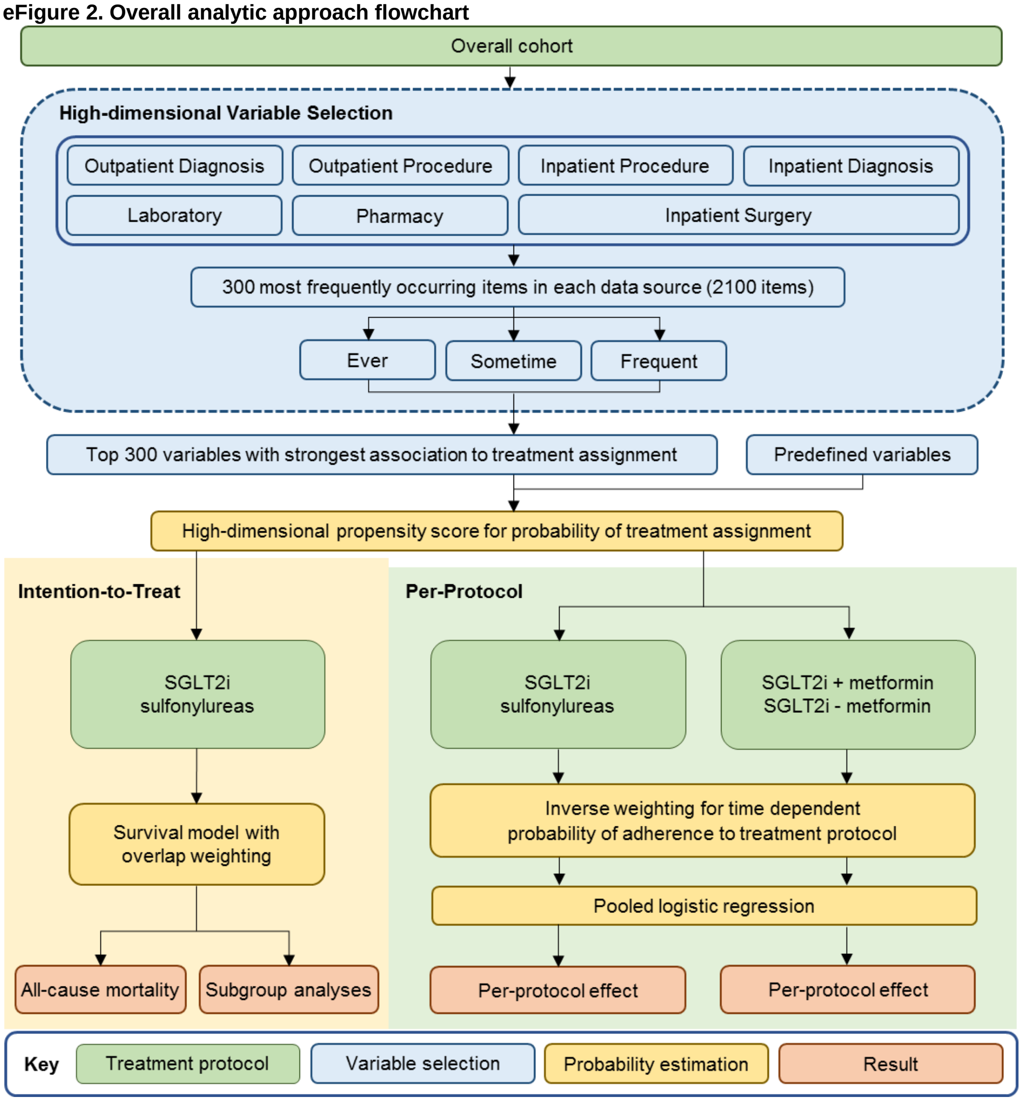

eFigure 3. Propensity score distribution of the SGLT2i and sulfonylurea arms

eFigure 3a. Propensity score distribution of the SGLT2i and sulfonylurea arms before adjustment

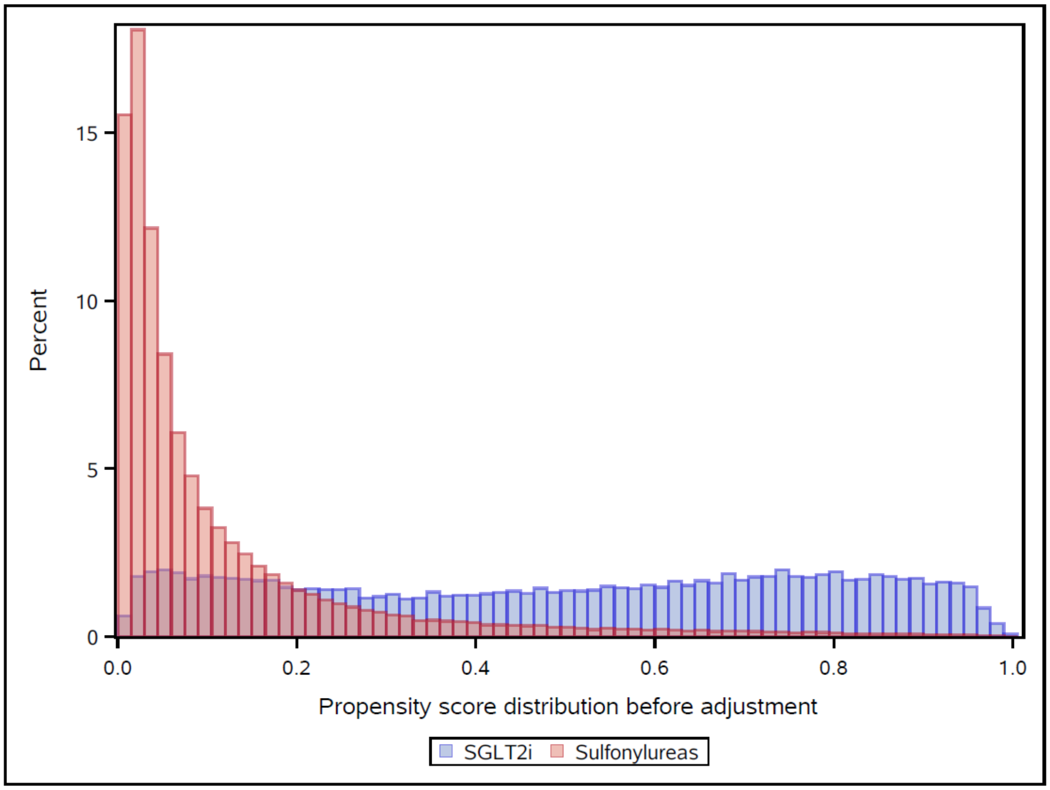

eFigure 3b. Propensity score distribution of the SGLT2i and sulfonylurea arms after adjustment

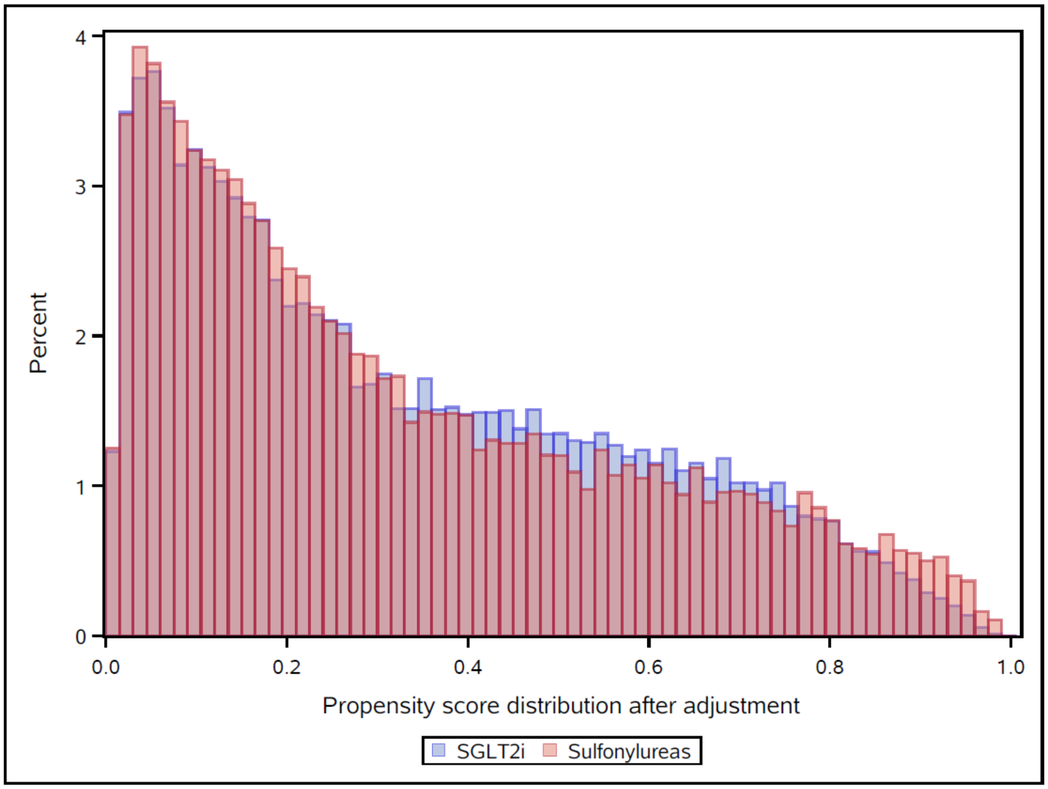

eFigure 4. Standardized differences between the SGLT2i and sulfonylurea arms before and after adjustment

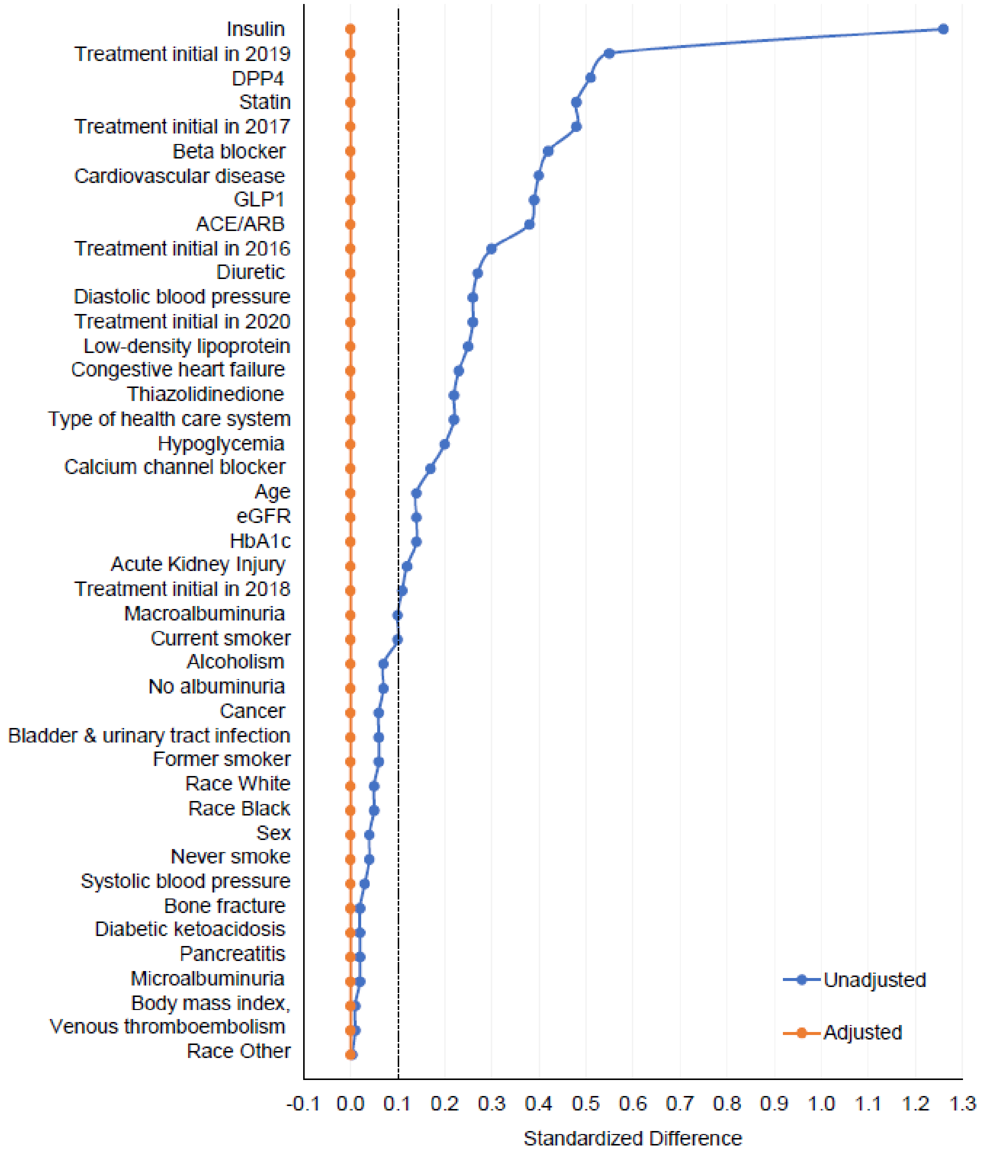

eFigure 5. Adjusted survival probability for negative and positive outcome controls

eFigure 5a. for chronic lower respiratory disease (negative outcome control). Survival probability in SGLT2i (blue) and sulfonylureas (red) arms, according to an intention-to-treat analysis. Light colored bands represent the 95% confidence interval for each treatment.

eFigure 5b. Adjusted survival probability for BMI decrease >10% (positive outcome control). Survival probability in SGLT2i (blue) and sulfonylureas (red) arms, according to an intention-to-treat analysis. Light colored bands represent the 95% confidence interval for each treatment.

Commented [AZ1]: Why is this page blank? Can we move the figure here?

Commented [AZ2]: Blank page?
Can we move the figure here?

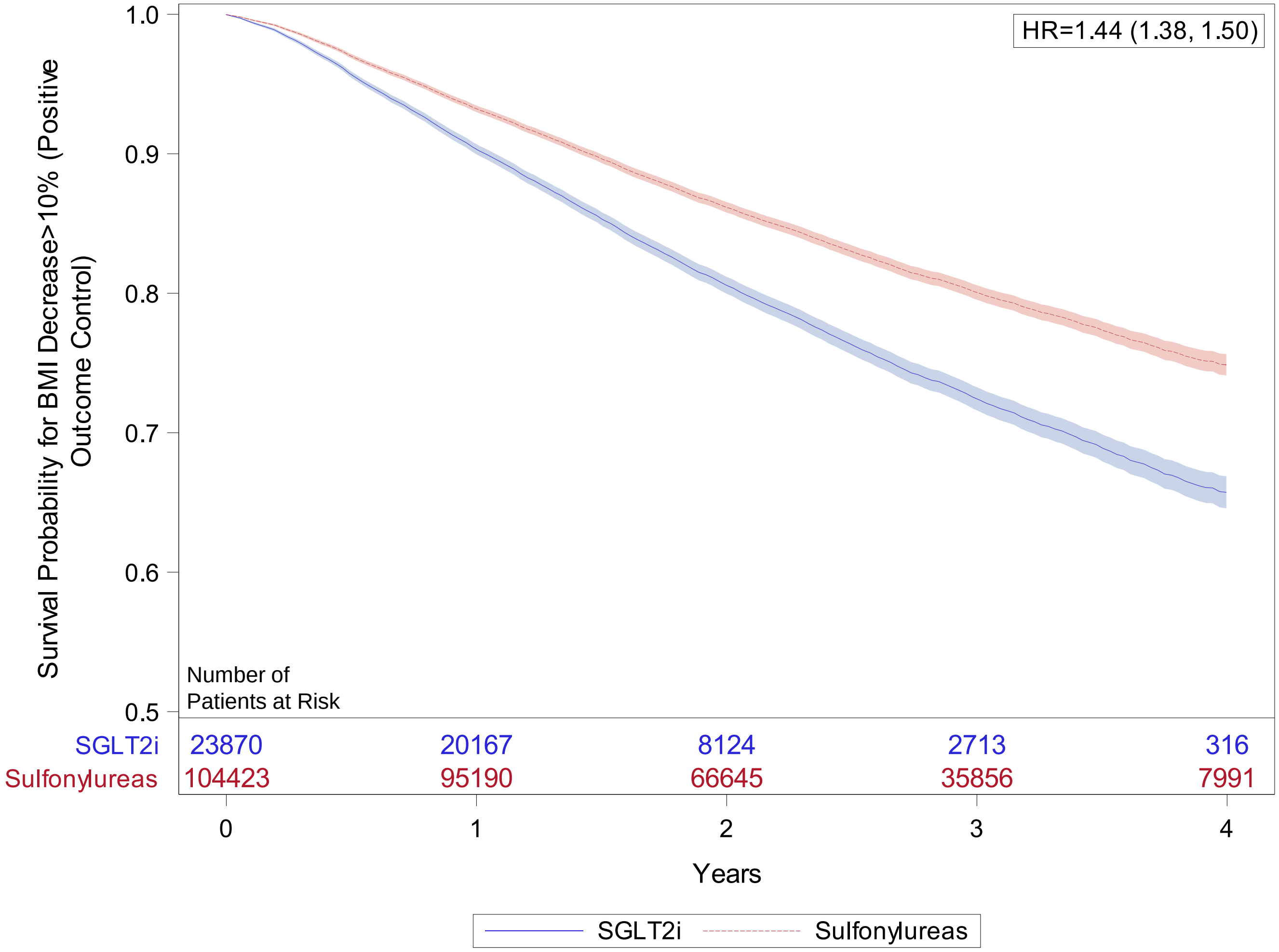

© 2021 Xie Y et al. *JAMA Internal Medicine*.

eFigure 5c. Adjusted survival probability for BMI increase >10% (positive outcome control). Survival probability in SGLT2i (blue) and sulfonylureas (red) arms, according to an intention-to-treat analysis. Light colored bands represent the 95% confidence interval for each treatment.

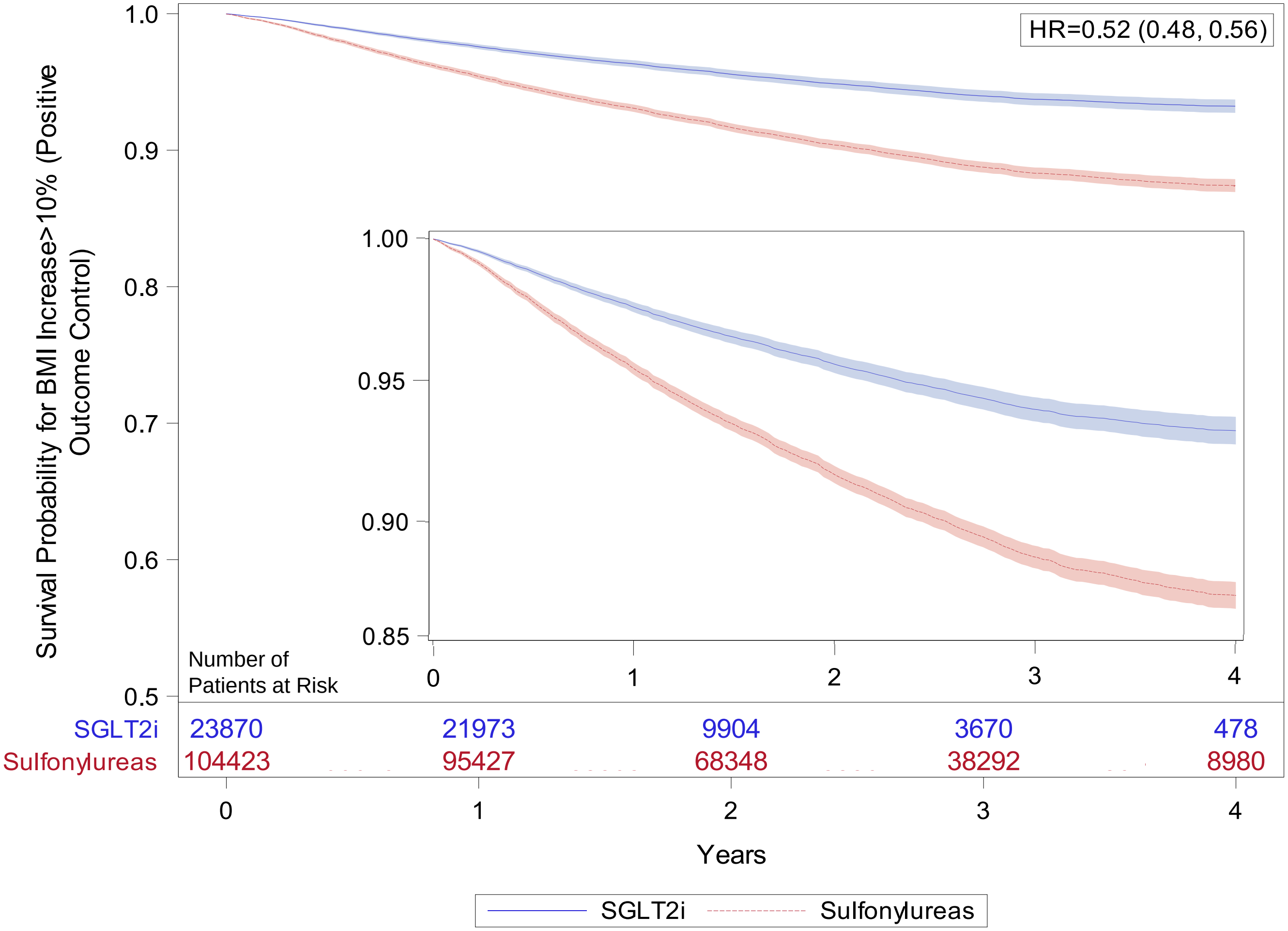

© 2021 Xie Y et al. *JAMA Internal Medicine*.

eTable 1. Distribution of medications within each antihyperglycemic class at treatment initiation

SGLT2i		Sulfonylureas	
**Medication**	**n (%)**	**Medication**	**n (%)**
Empagliflozin	23816 (99.77)	Glipizide	94856 (90.84)
Canagliflozin	38 (0.16)	Glimepiride	9124 (8.74)
Dapagliflozin	16 (0.07)	Glyburide	443 (0.42)

eTable 2. Demographic and health characteristics of the unadjusted cohort

Baseline Characteristics	Overall cohort, no. (%) (n=128293)	SGLT2i, no. (%) (n=23 870 [18.61%])	Sulfonylurea, no. (%) (n=104 423 [81.39%])	Absolute standardized difference
Age, mean (SD), years	63.84 (10.67)	65.00 (9.28)	63.57 (10.94)	0.14
Race				
White	95926 (74.77)	18283 (76.59)	77643 (74.35)	0.05
Black	25413 (19.81)	4312 (18.06)	21101 (20.21)	0.05
Other	6954 (5.42)	1275 (5.34)	5679 (5.44)	0.004
Sex				0.04
Male	121856 (94.98)	22825 (95.62)	99031 (94.84)	
Female	6437 (5.02)	1045 (4.38)	5392 (5.16)	
eGFR, mean (SD), mL/min/1.73 m^2	80.31 (19.79)	78.15 (18.38)	80.81 (20.07)	0.14
eGFR status				
≥ 90 mL/min/1.73 m^2	42905 (33.44)	6596 (27.63)	36309 (34.77)	0.15
≥ 60 to < 90 mL/min/1.73 m^2	63674 (49.63)	12928 (54.16)	50746 (48.60)	0.11
≥ 45 to > 60 mL/min/1.73 m^2	16623 (12.96)	3812 (15.97)	12811 (12.27)	0.11
30 to > 45 mL/min/1.73 m^2	5091 (3.97)	534 (2.24)	4557 (4.36)	0.12
HbA1c, mean (SD), %	8.77 (1.84)	8.58 (1.47)	8.82 (1.91)	0.14
Body mass index, mean (SD), kg/m^2	33.14 (6.58)	34.54 (6.59)	32.83 (6.54)	0.01
Low-density lipoprotein, mean (SD), mg/dL	87.82 (40.12)	79.95 (35.44)	89.62 (40.90)	0.25
Systolic blood pressure, mean (SD), mmHg	132.58 (16.66)	132.22 (16.53)	132.67 (16.69)	0.03
Diastolic blood pressure, mean (SD), mmHg	77.13 (10.29)	74.95 (9.99)	77.63 (10.29)	0.26
Congestive heart failure	7862 (6.13)	2653 (11.11)	5209 (4.99)	0.23
Alcoholism	7730 (6.03)	1118 (4.68)	6612 (6.33)	0.07
Bone fracture	1311 (1.02)	277 (1.16)	1034 (0.99)	0.02
Cancer	23446 (18.28)	4863 (20.37)	18583 (17.80)	0.06
Cardiovascular disease	30296 (23.61)	9066 (37.98)	21230 (20.33)	0.40
Diabetic ketoacidosis	305 (0.24)	75 (0.31)	230 (0.22)	0.02
Hypoglycemia	1723 (1.34)	908 (3.80)	815 (0.78)	0.20
Pancreatitis	1367 (1.07)	300 (1.26)	1067 (1.02)	0.02
Bladder & urinary tract infection	3303 (2.57)	453 (1.90)	2850 (2.73)	0.06
Venous thromboembolism	690 (0.54)	150 (0.63)	540 (0.52)	0.01
Acute Kidney Injury	8842 (6.89)	2296 (9.62)	6546 (6.27)	0.12
Albuminuria [a]				
No albuminuria	76121 (59.33)	13481 (56.48)	62640 (59.99)	0.07
Microalbuminuria	44545 (34.72)	8481 (35.53)	36064 (34.54)	0.02
Macroalbuminuria	7627 (5.94)	1908 (7.99)	5719 (5.48)	0.10
Insulin	31818 (24.80)	16093 (67.42)	15725 (15.06)	1.26
DPP4	10176 (7.93)	4290 (17.97)	5886 (5.64)	0.51
GLP1	4801 (3.74)	3499 (14.66)	1302 (1.25)	0.39
Thiazolidinedione	2799 (2.18)	1286 (5.39)	1513 (1.45)	0.22
ACE/ARB	70989 (55.33)	16781 (70.30)	54208 (51.91)	0.38
Calcium channel blocker	32153 (25.06)	7425 (31.11)	24728 (23.68)	0.17
Beta blocker	47107 (36.72)	12679 (53.12)	34428 (32.97)	0.42
Diuretic	45576 (35.52)	10983 (46.01)	34593 (33.13)	0.27
Statin	88781 (69.20)	20385 (85.40)	68396 (65.50)	0.48
Type of health care system				0.22
Outpatient clinic	79769 (62.18)	12294 (51.50)	67475 (64.62)	
Hospital system	48524 (37.82)	11576 (48.50)	36948 (35.38)	
Year of treatment initial				
2016	8517 (6.64)	379 (1.59)	8138 (7.79)	0.30
2017	39915 (31.11)	3490 (14.62)	36425 (34.88)	0.48
2018	37619 (29.32)	6038 (25.30)	31581 (30.24)	0.11
2019	37442 (29.18)	11920 (49.94)	25522 (24.44)	0.55

2020	4800 (3.74)	2043 (8.56)	2757 (2.64)	0.26
Smoking status				
Never	68740 (53.59)	13131 (55.01)	55609 (53.25)	0.04
Former	31121 (24.26)	6260 (26.23)	24861 (23.81)	0.06
Current	28432 (22.16)	4479 (18.76)	23953 (22.94)	0.10
Follow up, mean (SD), years	2.57 (1.00)	2.04 (0.86)	2.70 (0.99)	0.71

Abbreviations: SGLT2i, sodium-glucose co-transporter-2 inhibitor; SD, standard deviation; eGFR, estimated glomerular filtration rate; HbA1c, glycated hemoglobin; DPP4, dipeptidyl peptidase-4 inhibitor; GLP1, glucagon-like peptide-1 receptor agonist; ACE/ARB, angiotensin converting enzyme inhibitors/angiotensin-receptor blockers.

[a] No albuminuria defined as albumin to creatinine ratio < 30 mg/g, microalbuminuria defined as albumin to creatinine ratio from 30 to < 300 mg/g, and macroalbuminuria defined as albumin to creatinine ratio of ≥ 300 mg/g.

eTable 3. Intention-to-treat hazard ratios, death rates, and event reductions in the overall cohort and in subgroups

Analyses	Adjusted hazard ratio (95%CI)	Adjusted death rate in SGLT2i per 1000 person-years	Adjusted death rate in sulfonylurea per 1000 person-years	Event reduction per 1000 person-years
Overall	0.81 (0.75, 0.87)	23.01 (21.48, 24.8)	28.23 (27.1, 29.42)	-5.15 (-7.16, -3.02)
Age				
≤65	0.85 (0.73, 0.99)	12.31 (10.7, 14.11)	14.49 (13.19, 15.93)	-2.17 (-4.32, -0.29)
>65	0.81 (0.74, 0.88)	31.58 (29.19, 33.87)	38.69 (36.53, 40.15)	-7.05 (-9.71, -3.99)
CVD				
No	0.81 (0.75, 0.88)	23.13 (21.65, 24.49)	28.06 (27.02, 29.47)	-4.95 (-6.90, -3.31)
Yes	0.85 (0.75, 0.97)	21.4 (19.1, 23.88)	24.98 (23.34, 26.94)	-3.59 (-6.14, -0.89)
eGFR, mL/min/1.73 m^2				
≥90	0.79 (0.67, 0.94)	14.01 (11.86, 16.85)	17.81 (15.98, 19.12)	-3.74 (-6.2, -0.68)
≥60-<90	0.86 (0.78, 0.96)	24.6 (22.51, 26.75)	28.17 (26.47, 29.66)	-3.51 (-6.19, -0.99)
≥45-<60	0.78 (0.66, 0.91)	32.78 (28.37, 37.2)	41.72 (38.13, 45.18)	-9.18 (-13.53, -2.91)
≥30-<45	0.71 (0.5, 1.00)	47.63 (32.64, 62.88)	64.67 (54.6, 75.96)	-18.27 (-33.34, -1.1)
Albuminuria status [a]				
No albuminuria	0.88 (0.79, 0.98)	19.75 (17.93, 21.65)	22.4 (21.15, 23.62)	-2.71 (-4.69, -0.79)
Micro-albuminuria	0.79 (0.7, 0.89)	26.88 (24.19, 29.38)	33.36 (31.26, 35.72)	-6.69 (-10.02, -3.48)
Macro-albuminuria	0.68 (0.53, 0.85)	33.63 (27.11, 40.67)	48.44 (41.35, 54.32)	-14.70 (-23.04, -5.97)
BMI, kg/m^2				
>18.5- ≤25	0.69 (0.54, 0.87)	42.05 (32.97, 50.44)	59.27 (52.27, 65.78)	-17.40 (-28.04, -7.58)
>25- ≤30	0.84 (0.73, 0.97)	26.07 (23.2, 29.02)	30.64 (28.22, 33.16)	-4.64 (-7.8, -1.15)
>30	0.84 (0.77, 0.92)	20.86 (19.4, 22.49)	24.67 (23.3, 26.08)	-3.73 (-5.56, -1.95)
Insulin				
No	0.84 (0.75, 0.95)	18.58 (16.37, 20.56)	21.84 (20.83, 23.08)	-3.36 (-5.45, -0.71)
Yes	0.79 (0.72, 0.87)	26.92 (25.17, 29.09)	33.54 (31.64, 35.8)	-6.57 (-9.4, -3.9)
ACE/ARB				
No	0.85 (0.75, 0.97)	21.4 (19.1, 23.88)	24.98 (23.34, 26.94)	-3.59 (-6.14, -0.89)
Yes	0.79 (0.73, 0.87)	24.04 (22.31, 26.21)	29.88 (28.18, 31.65)	-5.81 (-7.84, -3.66)
Diuretic				
No	0.86 (0.78, 0.96)	18.97 (17, 20.71)	21.97 (20.74, 23.21)	-3.10 (-5.17, -0.67)
Yes	0.77 (0.70, 0.86)	28.94 (26.74, 31.69)	37.2 (34.83, 39.82)	-8.26 (-11.24, -4.79)

Abbreviations: SGLT2i, sodium-glucose co-transporter-2 inhibitor; CI, confidence interval; CVD, cardiovascular disease; eGFR, estimated glomerular filtration rate; BMI, body mass index; ACE/ARB, angiotensin converting enzyme inhibitors/angiotensin-receptor blockers.

[a] Albuminuria status defined as: no albuminuria (albumin to creatinine ratio≤30 mg/g), microalbuminuria (>30 to ≤300 mg/g), and macroalbuminuria (>300 mg/g).

eTable 4. Adherence rate, estimated event rate, adjusted hazard ratio, and estimated event reduction for all-cause mortality based on per-protocol analyses for continued use of SGLT2i or sulfonylureas

Treatment groups	Adherence rate [a]	Estimated event rate per 1000 person-years	Adjusted hazard ratio (95% CI)	Event reduction per 1000 person-years (95% CI)
SGLT2i (N=23870)	51.80%	21.18 (18.84, 24.08)	0.66 (0.60, 0.74)	-10.10 (-12.97, -7.24)
Sulfonylureas (N=104423)	39.69%	32.22 (27.91, 35.47)		

Abbreviations: SGLT2i, sodium-glucose co-transporter-2 inhibitor; CI, confidence interval.
[a] Adherence rate based on proportion of participants in the treatment group adherence to the protocol at the end of follow up, after adjustment of baseline characteristics

eTable 5. Adherence rate, estimated event rate, adjusted hazard ratio, and estimated event reduction for all-cause mortality based on per-protocol analyses for continued use of SGLT2i with and without metformin

Treatment groups	Adherence rate [a]	Estimated event rate per 1000 person-years	Adjusted hazard ratio (95% CI)	Event reduction per 1000 person-years (95% CI)
SGLT2i with metformin (N=22542)	36.28%	18.48 (16.01, 22.09)	0.70 (0.50, 0.97)	-7.62 (-17.12, -0.48)
SGLT2i without metformin (N=1328)	25.57%	26.26 (18.48, 36.40)		

Abbreviations: SGLT2i, sodium-glucose co-transporter-2 inhibitor; CI, confidence interval.
[a] Adherence rate based on proportion of participants in the treatment group adherence to the protocol at the end of follow up, after adjustment of baseline characteristics
